# Roles of follicle stimulating hormone and sphingosine 1-phosphate co-administered in the process in mouse ovarian vitrification and transplantation

**DOI:** 10.1186/s13048-023-01206-1

**Published:** 2023-08-24

**Authors:** Fei Wang, Yuan Tian, Liwen Huang, Tian Qin, Wenye Ma, Chengbin Pei, Bo Xu, Hang Han, Xinrui Liu, Pengge Pan, Xiaoli Yu, Qin Chang, Yanrong Wang, Shuya Zhang, Xiuying Pei

**Affiliations:** 1 Key Laboratory of Fertility Preservation and Maintenance of Ministry of Education, School of Basic Medical Sciences, Ningxia Medical University, Yinchuan, Ningxia 750004 China; 2 General Hospital of Ningxia Medical University, Yinchuan, Ningxia China; 3 Centre of Assisted Reproduction, Maternal and Children Health Care Hospital of Yinchuan, Yinchuan, China

**Keywords:** Ovarian tissue, FSH, S1P, Vitrification, Transplantation

## Abstract

Some major challenges of ovarian tissue vitrification and transplantation include follicle apoptosis induced by cryopreservation and ischemia-reperfusion injury, as well as ovarian follicle loss during post-transplantation. This research aimed to investigate the protective effects and underlying mechanisms of follicle-stimulating hormone (FSH) and Sphingosine-1-phosphate (S1P) on vitrified and post-transplantation ovaries. Ovaries from 21-day-old mice were cryopreservation by vitrification with 0.3 IU/mL FSH, 2 µM S1P, and 0.3 IU/mL FSH + 2 µM S1P, respectively, for follicle counting and detection of apoptosis-related indicators. The results demonstrated that FSH and S1P co-intervention during the vitrification process could preserve the primordial follicle pool and inhibit follicular atresia by suppressing cell apoptosis. The thawed ovaries were transplanted under the renal capsule of 6–8 week-old ovariectomized mice and removed 24 h or 7 days after transplantation. The results indicated that FSH and S1P co-intervention can inhibit apoptosis and autophagy in ovaries at 24 h after transplantation, and promote follicle survival by up-regulating Cx37 and Cx43 expression, enhanced angiogenesis in transplanted ovaries by promoting VEGF expression, as well as increased the E_2_ levels to restore ovarian endocrine function at 7 days after transplantation. The hypoxia and ischemia cell model was established by CoCl_2_ treatment for hypoxia in human granulosa-like tumor cell line (KGN), as well as serum-free culture system was used for ischemia. The results confirmed that ischemia-hypoxia-induced apoptosis in ovarian granulosa cells was reduced by FSH and S1P co-intervention, and granulosa cell autophagy was inhibited by up-regulating the AKT/mTOR signaling pathway. In summary, co-administration of FSH and S1P can maintain ovarian survival during ovarian vitrification and increase follicle survival and angiogenesis after transplantation.

**Supplementary Information:**

The online version contains supplementary material available at 10.1186/s13048-023-01206-1.

## Introduction

Advances in cancer treatment have extended the life expectancy of women with cancer. However, chemotherapy can cause irreversible ovarian damage [1], reduce primordial follicular reserve [2, 3], and cause premature ovarian failure (POF) [4]. Ovarian cryopreservation by vitrification and transplantation technology has been shown to be an effective method for restoring ovarian endocrine function, preserving a large number of primordial follicles, and allowing natural pregnancy without delaying treatment of cancer patients [5–9].

This technology provides hope to female cancer patients who desire to maintain fertility [10]. Unfortunately, apoptosis of follicles induced by cryopreservation [11], massive loss of primordial follicles due to early ischemia-reperfusion injury after transplantation without surgical vascular anastomosis [12], and apoptosis of oocytes and granulosa cells due to oxidative stress [13] are the main bottlenecks of this technology. Therefore, reducing the freezing damage during ovarian cryopreservation, increasing the follicle survival rate, and improving anti-apoptosis and angiogenesis in post-transplant ovaries are key issues that need to be addressed.

S1P is a sphingolipid metabolite produced in cells by the action of sphingosine kinase-1 (SK-1) and sphingosine kinase-2 (SK-2) [14, 15]. S1P can interact as a first messenger with G protein-coupled receptors (GPCRs) on the cell membrane to participate in biological activities including cell migration, survival, proliferation, angiogenesis, immunity, and allergic reactions [16–18]. S1P has been widely used in the field of reproduction. S1P has been shown to inhibit granulosa cell apoptosis in ovaries and promote ovarian autoangiogenesis [19]. Furthermore, exogenous administration of S1P was shown to protect the ovarian follicle reserve by decreasing apoptosis during chemoradiotherapy [20–23], improve the survival rate of cryopreserved ovaries, increase the vascular density in ovarian grafts, and promote angiogenesis of the transplanted ovaries [24, 25]. Nevertheless, S1P has a short half-life and cannot maintain long-term effects in vitro culture [26]. Therefore, the development of measures to promote the continuous production of S1P in order to achieve its biological activity is a key imperative.

Studies have indicated that FSH can promote S1P production in ovarian granulosa cells [27]. Our previous research showed that FSH can enhance the blood supply of transplanted ovaries, improve the survival rate of follicles, and inhibit ovarian cell apoptosis [28, 29]. Since S1P cannot sustain its effects in vitro, we believe that co-administration of S1P and appropriate concentration of FSH stimulate endogenous S1P production and prolong its biological activity.

Recently, it has been shown that FSH can inhibit excessive autophagy in ovarian granulosa cells caused by oxidative stress injury by coordinating the PI3K-AKT-mTOR axis, PI3K- AKT-FOXO1 signaling cascade, and FOXO1 acetylation-dependent pathway [30]. Whereas, S1P is able to induce protective autophagy following increased SK-1 activity during nutrient starvation, thereby saving cells from cell death with apoptotic features [31], it is unknown whether autophagy is triggered by FSH and S1P to protect the survival of transplanted ovarian follicles. Therefore, this study will investigate the protective effect of FSH and S1P on follicle survival and angiogenesis of vitrification and transplanted ovarian tissue. Additionally, the granulosa cells suffered with ischemic and hypoxic in the early stage of ovarian transplantation in vitro, we also constructed granulosa cell model with ischemic and hypoxic state to investigate the molecular mechanism of FSH and S1P intervention to protect the survival of follicles in transplanted ovaries, so as to provide a theoretical basis for improving follicle survival in cryopreserved and post-transplant ovaries.

## Materials and methods

### Animals and treatments

All animal experimental procedures were approved by the Institutional Animal Care and Use Committee of the Ningxia Medical University. A total of 125 ICR female mice (Experimental Animal Center of the Ningxia Medical University) were housed in a controlled environment (temperature: 24 ± 2℃; humidity: 40–50%, 12 h light/12 h dark cycle) with free access to food and water. The mice were anesthetized by intraperitoneal injection of 1% sodium pentobarbital (0.01 mL/g bodyweight), seventy-five 21-day-old mice and randomly divided into 5 groups (n = 15 per group): (A) Fresh group (mice fresh ovaries); (B) PBS group (PBS was administered into the medium during the entire vitrification/thawing process); (C) FSH group (0.3 IU/mL FSH was administered into the medium during the entire vitrification/thawing process); (D) S1P group (2 µM S1P was administered into the medium during the entire vitrification/thawing process); (E) FSH + S1P group (0.3 IU/mL FSH and 2 µM S1P was administered into the medium during the entire vitrification/thawing process) for vitrification. Fifty 6–8 weeks old mice were used for heterotopic transplantation as recipient mice.

### Cell culture and treatment

The human granulosa cell line of KGN (Procell CL-0603) was obtained from Procell Life Technology Co. Ltd. KGN cells were cultured in DMEM/F12 (1:1) medium (Hyclone, SH30023.01) supplemented with 10% fetal bovine serum (FBS) (Biological Industries, 04-001AUS-1 A) with 100 units/mL penicillin and 100 µg/mL streptomycin (Solarbio, P1400) at 37 °C with 5% CO_2_. The groups were divided as follows: (A) Control group; (B) Vehicle group (The KGN cells were cultured with 400 µM CoCl_2_); (C) FSH group (The KGN cells were cultured with 2.4 IU/mL FSH and 400 µM CoCl_2_); (D) S1P group (The KGN cells were cultured with 2 µM S1P and 400 µM CoCl_2_); (E) FSH + S1P group (The KGN cells were cultured with 2.4 IU/mL FSH, 2 µM S1P and 400 µM CoCl_2_). KGN cells were exposed to 400 µM CoCl_2_ (Sigma-Aldrich, 255599) for 1 h in the serum-free medium when the cells grew to 60-70%, and cultured with serum-free DMEM/F-12 containing 2.4 IU/mL FSH, 2 µM S1P (Sigma-Aldrich, 26993-30-6) and 100 nM Rapamycin (Beyotime Biotechnology, SF2681) for 24 h as indicated, respectively, CCK-8 was used for cell viability assay, TUNEL method was used for cell apoptosis assay, and cells were transfected with MAP1LC3B expression plasmid (GFP-MAP1LC3B) for autophagosome formation assay.

### Ovarian vitrification and transplantation

The preparation of the vitrification and thawing solution is provided in the supplementary data. Briefly, the ovaries were precultured in culture solution for 1 h at 37℃ with 5% CO_2_ and then were equilibrated for 7 min. Next, the ovaries were then immersed in vitrification solution for 3 min and then quickly placed in liquid nitrogen for storage. After one week of cryopreservation, the ovaries were thawed for 10 min with a gradient thawing solution and postcultured in culture solution for 2 h at 37℃ with 5% CO_2_. The ovary transplantation procedures were performed according to previously described [28]. The dorsolateral skin of recipient mice was shaved and sterilized after anesthesia, and then the bilateral ovaries were removed through small dorsolateral skin incisions, and the kidneys were identified on both sides of the spine and fixed. A small opening was poked with forceps in the renal capsule, and the thawed ovary was inserted by the small opening into the renal capsule. Then the kidneys were reset and sutured. The grafts were removed for subsequent experiments 24 h and 7 days after transplantation.

### Follicle count

The paraffin-embedded ovaries were cut into serial sections and then one of every five sections was selected for HE-staining (Solarbio, G1121). Follicle count was done according to previous studies [32, 33]. The criterion of the follicle count was done as follows, total number of follicles = normal follicles + atretic follicles; percentage of follicles at each stage = total number of follicles at each stage/total number of follicles.

### Vascular perfusion

7 days after ovarian transplantation, mice were injected with 2MDa-FITC-dextran (Sigma, 60842-46-8) 0.1 mL through the tail vein. An hour later, the kidneys were made into frozen sections with ovary grafts. The frozen sections were used for immunofluorescence staining of CD31 (Abcam, ab28364, 1:50) and analyzed the positive area using Image-Pro Plus 6.0 software. The ratio of CD31 positive area to total ovarian area represents vascular density. The ratio of CD31 positive area to FITC-dextran positive area represents the vascular area with perfusion function.

### Western blot analysis

The ovaries and cells were lysed with lysis buffer, then the protein concentration was determined and quantified by the BCA assay (KeyGEN BioTECH, KGPBCA). The protein samples were transferred onto the polyvinylidene difluoride (PVDF) membranes after SDS-PAGE gel electrophoresis. The membranes were blocked with 5% nonfat milk for 1 h, then probed with primary antibodies overnight at 4℃, and the dilution method of the primary antibody is provided in the supplementary data. Next, the membranes were washed with TBST buffer and incubated with HRP-conjugated IgG secondary antibody (Rabbit:ZSGB-BIO, ZB-2301, 1:10,000) (Mouse: ZSGB-BIO, ZB-2305, 1:10,000) for 1 h at room temperature. The resulting signal was assayed with ECL kit (KeyGEN BioTECH, KGP1127) and photographed in Bio-Rad’s ChemiDOXTMXRS+Chemiluminescence systems with exposure. β-actin was used as the reference Protein. The results were analyzed using the software Image J.

### Statistical analysis

All experiments were performed at least 3 times. Data analyses were performed using SPSS 17.0 and Graph Pad Prism 8 software (Graph Pad). Continuous variables were expressed as mean ± standard error of the mean (SEM) and between-group differences were assessed using Student’s *t* test. *P* values < 0.05 were considered indicative of statistical significance.

## Results

### FSH combined with S1P protects the ovaries from vitrification-induced damage

**Fig. 1:**
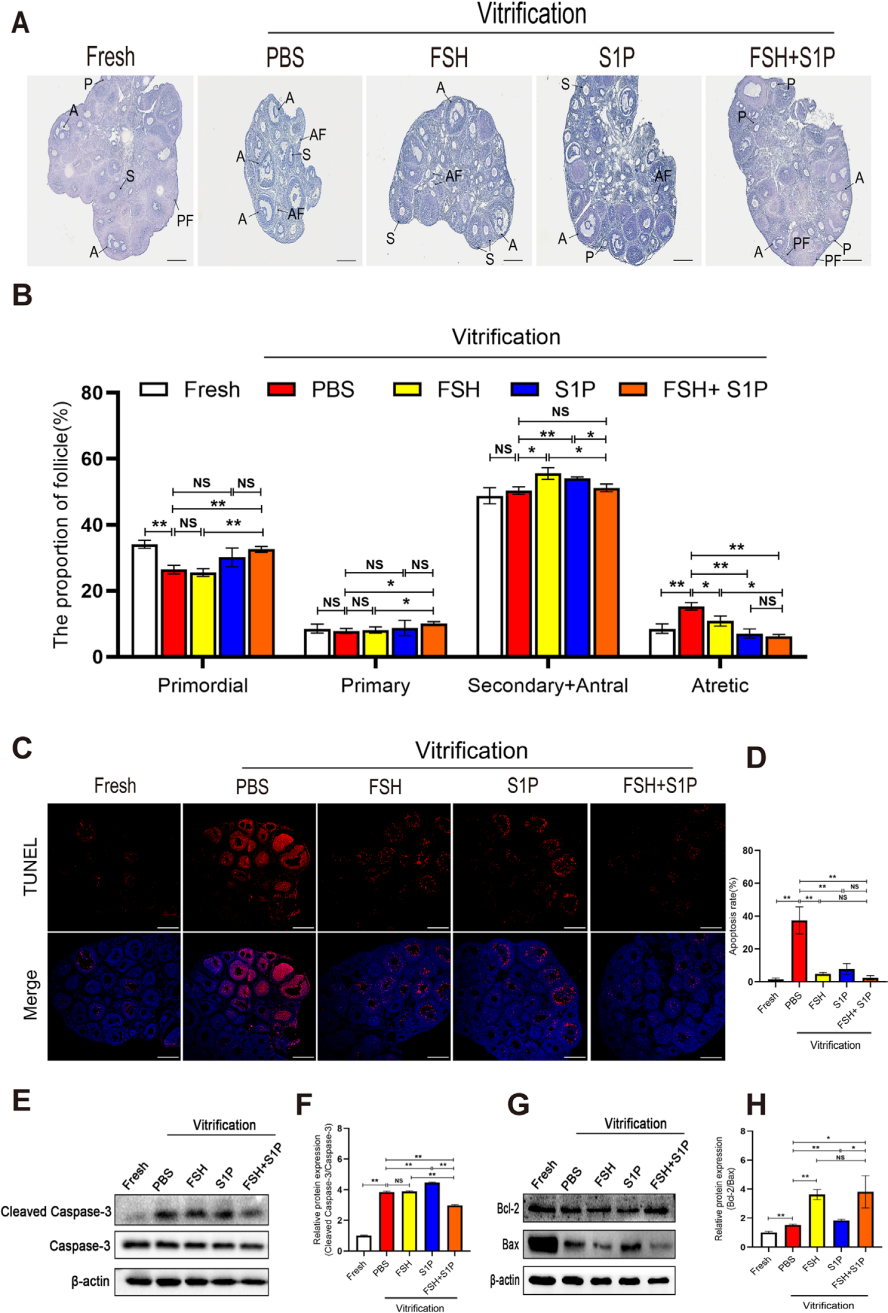
**FSH combined with S1P protects ovaries from vitrification-induced damage** (**A**) HE-stained sections of vitrified-thawed ovaries (Scale bar: 200 μm). (**B**) Follicle count at each stage (PF: primordial follicles; P: primary follicles; S: secondary follicles; A: antral follicles; AF: atretic follicles). (**C**-**D**) TUNEL staining to detect apoptosis of vitrified-thawed ovaries (Scale bar: 200 μm) and statistical analysis of apoptotic rate. (**E**-**F**) Western blot analysis of the expression of Cleaved Caspase-3 in vitrified-thawed ovaries and its quantification. (**G**-**H**) Western blot analysis of the expression of Bcl-2 and Bax in vitrified-thawed ovaries and quantification of Bcl-2/Bax ratio. (*P < 0.05; **P < 0.01)

To explore the protective effect of the FSH and S1P on ovarian follicle survival of vitrified-thawed ovaries, we performed follicle count by using HE-staining (Fig. 1A, B), and results revealed that the proportion of primordial follicles and primary follicles in the FSH + S1P group was significantly higher than that in the PBS group and the FSH group (*P* < 0.05), but there was no significant difference between the FSH + S1P group and the S1P group in this respect (*P* > 0.05). The proportion of secondary and antral follicles in the FSH + S1P group was lower than that in the FSH group and S1P group (*P* < 0.05). Meanwhile, the proportion of atretic follicles in the FSH + S1P group was lower than that in the PBS group and FSH group (*P* < 0.05), but there was no significant difference with the S1P group (*P* > 0.05).

To analyze whether the co-intervention of FSH and S1P protects ovaries from vitrification-induced damage by inhibiting apoptosis, we performed TUNEL staining and detected apoptosis-related proteins. TUNEL positive staining was mainly located in granulosa cells of secondary follicles, antral follicles, and atresia follicles. By counting the apoptotic rate of ovaries, we found the apoptotic rate of the FSH + S1P group was significantly lower than that of the PBS group (*P* < 0.01), however, the effect of inhibiting apoptosis in the FSH + S1P group was not significantly different from that in the FSH group and S1P group (*P* > 0.05) (Fig. 1C, D). Furthermore, we examined the level of Cleaved Caspase-3, Caspase-3, Bcl-2, Bax protein expression by Western blot. The expression of Cleaved Caspase-3 was significantly increased in the PBS group compared with the Fresh group (*P* < 0.01), demonstrating that vitrification can cause ovarian cells apoptosis, however, the level of Cleaved Caspase-3 in the FSH + S1P group was remarkably lower than that in the PBS, FSH, and S1P groups (*P* < 0.01) (Fig. 1E, F). In addition, the Bcl-2/Bax ratio in the FSH + S1P group was significantly higher than that in the PBS and S1P groups (*P* < 0.05), but there was no significant difference with the FSH group (*P* > 0.05) (Fig. 1G, H). Collectively, these results indicated that the combined intervention of FSH and S1P can maintain the primordial follicle pool in vitrification ovaries and protect ovaries from vitrification-induced damage by inhibiting apoptosis, thereby decrease follicular atresia.

### FSH combined with S1P protects the ovaries from ischemia-hypoxia injury 24 h after transplantation

**Fig. 2:**
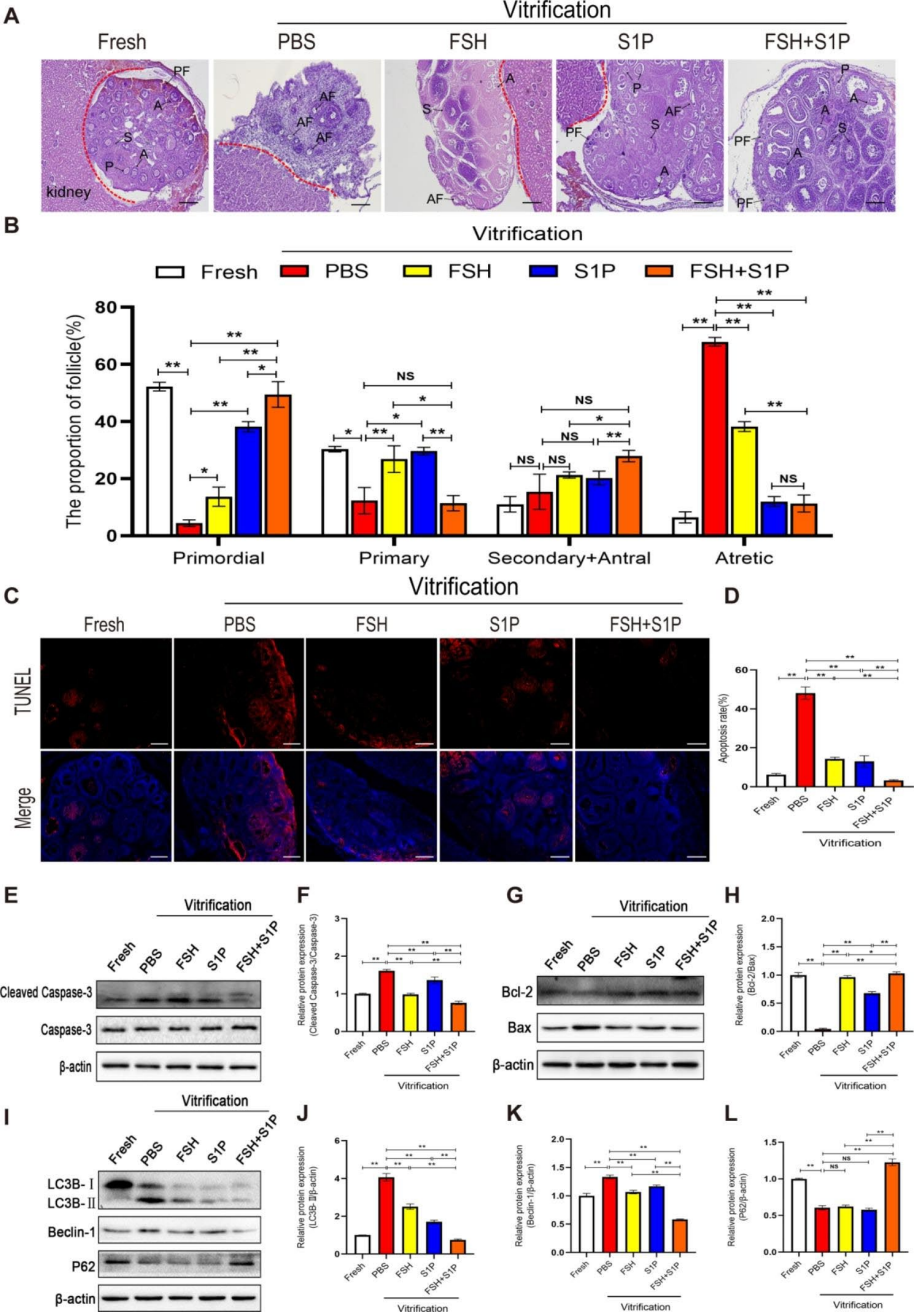
FSH combined with S1P protects the ovaries from ischemia-hypoxia injury at 24 h after transplantation (**A**) HE-stained sections of ovaries at 24 h after transplantation (Scale bar: 200 μm). (**B**) Follicle count at each stage (PF: primordial follicles; P: primary follicles; S: secondary follicles; A: antral follicles; AF: atretic follicles). (**C**-**D**) TUNEL staining to detect ovarian apoptosis at 24 h after transplantation (Scale bar: 200 μm) and statistical analysis of apoptotic rate. (**E**-**F**) Western blot analysis of the expression of Cleaved Caspase-3 of ovaries at 24 h after transplantation and its quantification. (**G**-**H**) Western blot analysis of the expression of Bcl-2 and Bax of ovaries at 24 h after transplantation and quantification of Bcl-2/Bax ratio. (I-L)Western blot analysis of the expression of LC3B, Beclin-1 and P62 of ovaries at 24 h after transplantation and its quantification. (*P < 0.05; **P < 0.01)

To clarify whether FSH and S1P have a protective effect on transplanted ovaries, we transplanted the vitrified-thawed ovaries under the renal capsule of 6–8 week old ovariectomized mice. After 24 h, we removed the grafts to perform follicle count (Fig. 2A, B) and detect the apoptosis and autophagy of transplanted ovaries. We found that the proportion of primordial follicles in the FSH + S1P group was approximately 50%, which was significantly greater than that in the PBS, FSH, and S1P groups (*P* < 0.05). However, the proportion of primary follicles in the FSH + S1P group was lower than that in the FSH and S1P groups (*P* < 0.05). The proportion of secondary and antral follicles in the FSH + S1P group was significantly higher than that in the FSH group and S1P group (*P* < 0.05). Furthermore, the proportion of atretic follicles in the FSH + S1P group was remarkably lower than that in the PBS and FSH groups (*P* < 0.01), but there was no significant difference with the S1P group (*P* > 0.05) (Fig. 2B). TUNEL staining analysis revealed that apoptosis occurred mainly in granulosa cells of secondary follicles, antral follicles, and atretic follicles, while it was rare in primordial follicles (Fig. 2C). We found follicle apoptosis was inhibited in the FSH, S1P, and FSH + S1P groups compared with the PBS group (*P* < 0.01); among these, the most significant inhibitory effect was observed in the FSH + S1P group, and there was a significant difference between the FSH and S1P groups in this respect (*P* < 0.01) (Fig. 2D). The expression of Cleaved Caspase-3 protein was also diminished in the FSH + S1P group compared with the PBS group, FSH group, and S1P group (*P* < 0.01) (Fig. 2E, F). Furthermore, the Bcl-2/Bax ratio in the FSH + S1P group was obviously higher than that in the PBS, FSH and S1P groups (*P* < 0.05) (Fig. 2G, H).

To explore whether FSH and S1P co-administration can inhibit the excessive autophagy in ovaries caused by ischemia-hypoxic stress in the early stage of transplantation, we detected the expression of several autophagy-related proteins in the ovaries at 24 h after transplantation (Fig. 2I). Our results showed that the expression of autophagic proteins (LC3B-II, Beclin-1) was significantly down-regulated in the transplanted ovaries of the FSH + S1P group compared with other groups (*P* < 0.01) (Fig. 2J, K). Moreover, the expression of P62 protein was the highest in the FSH + S1P group, which was remarkably higher than that in the PBS, FSH, S1P groups(*P* < 0.01). Taken together, these results demonstrated that FSH and S1P co-administration can reduce ovarian apoptosis and excessive autophagy caused by ischemia-hypoxia at 24 h after transplantation, inhibit follicle atresia, maintain a certain proportion of primordial follicle pool, and avoid excessive activation of primordial follicles.

### FSH combined with S1P promotes follicular survival and angiogenesis of ovaries at 7 days after transplantation

**Fig. 3:**
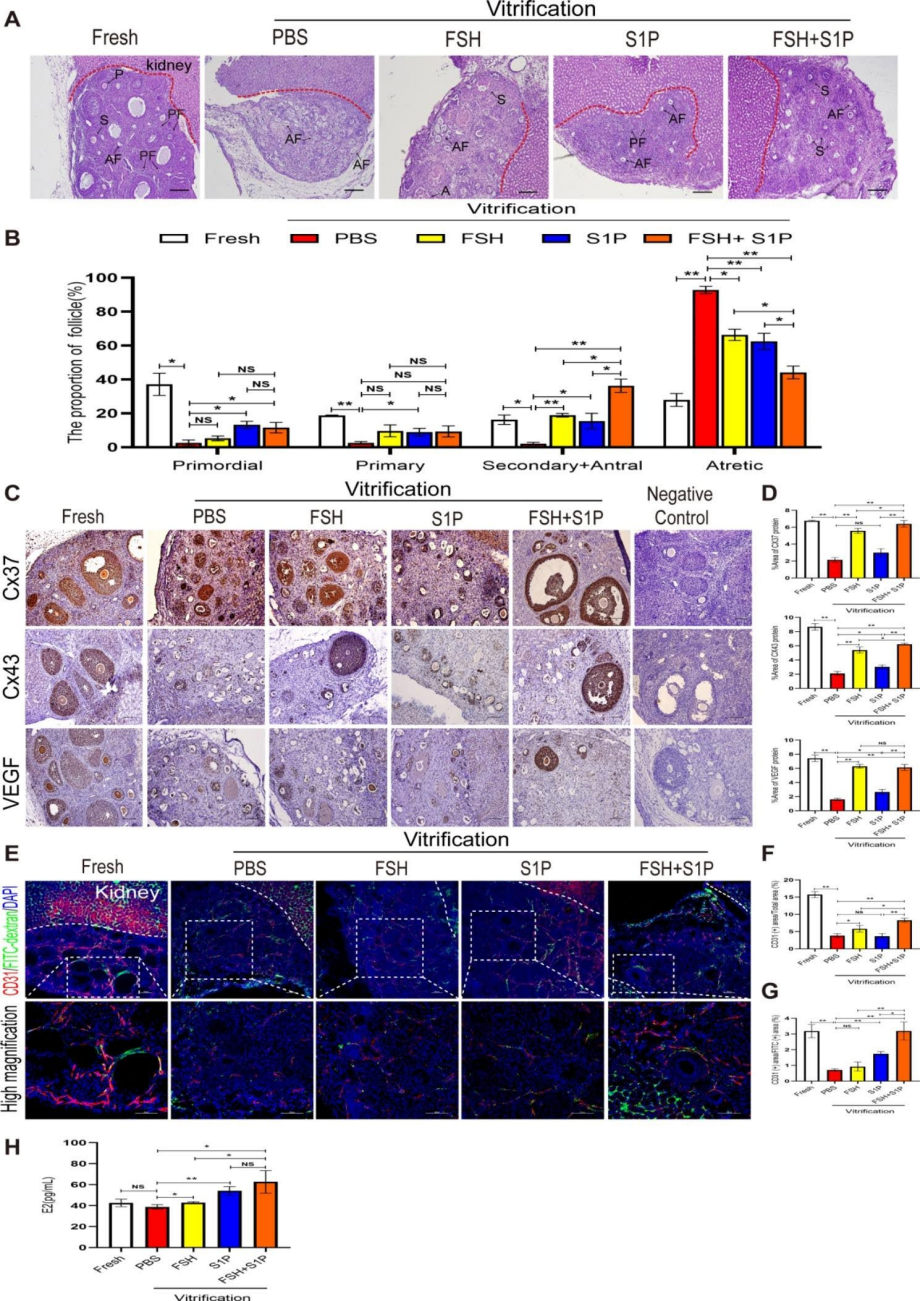
FSH combined with S1P promotes follicle survival and angiogenesis of ovaries at 7 days after transplantation (**A**) HE-stained sections of ovaries at 7 days after transplantation (Scale bar: 200 μm). (**B**) Follicle count at each stage (PF: primordial follicles; P: primary follicles; S: secondary follicles; A: antral follicles; AF: atretic follicles). (**C**-**D**) Immunohistochemical analysis of the expression of Cx37, Cx43, and VEGF, and statistical analysis of the respective positive areas (Scale bar:100 μm). (**E**) The graft angiogenesis is visualized by injecting the mice with 2 MDa FITC-dextran. The merged images of CD31 (red) and FITC-dextran angiography (green) were obtained by the confocal laser scanning microscope (Scale bar: 200 μm; high magnification: 100 μm). (**F**) Percentage of CD31-positive area of ovaries to the total area at 7 days after transplantation. (**G**) Percentage of CD31-positive area of ovaries to FITC-dextran positive area at 7 days after transplantation. (**H**) Results of ELISA showing the serum levels of E_2_ of mice at 7 days after transplantation. (*P < 0.05; **P < 0.01)

To assess whether FSH and S1P co-intervention can promote follicle survival and development in heterotopic transplanted ovaries, we removed the ovarian grafts at 7 days after transplantation to performed follicle count by HE-staining (Fig. 3A, B) and analyzed Cx37, Cx43, VEGF expression by immunohistochemistry (Fig. 3C, D). Based on the follicle count, the proportion of primordial follicles in the FSH + S1P group was prominently increased compared with the PBS group (*P* < 0.05), but there was no significant difference with the FSH and the S1P groups (*P* > 0.05), and the proportion of primary follicles in the FSH + S1P group was not significantly different from other groups (*P* > 0.05). Moreover, the proportion of secondary and antral follicles in the FSH + S1P group was significantly higher than that in the PBS, FSH, and S1P groups (*P* < 0.05), while the proportion of atretic follicles in the FSH + S1P group was significantly lower than that in the PBS, FSH and S1P groups (*P* < 0.05) (Fig. 3B). Cx37, Cx43, and VEGF are mainly expressed in the oocytes and granulosa cells of primordial, primary, secondary and antral follicles (Fig. 3C), the expression of Cx37 and Cx43 were significantly increased in the FSH + S1P group compared with the PBS, FSH, and S1P groups (*P* < 0.05). The expression of VEGF was also increased in the FSH + S1P group compared with the PBS and S1P groups (*P* < 0.05), but there was no significant difference with the FSH group (Fig. 3D).

To investigate the angiogenesis and perfusion in transplanted ovaries, we visualized capillaries and microvascular networks by injecting mice with 2MDa-FITC-dextran via the tail vein and detected the vascular density by the immunofluorescence of CD31 (Fig. 3E). We observed that the expression of the CD31 positive area in the FSH + S1P group was higher than that in the PBS, FSH, and S1P groups (*P* < 0.05) (Fig. 3F). In addition, the yellow area where FITC-dextran green fluorescence overlaps with CD31 red fluorescence is the blood vessel area with perfusion function: the expression of the CD31(+) area/FITC-dextran (+) area in the FSH + S1P group was significantly higher than that in the PBS, FSH, and S1P groups (*P* < 0.05) (Fig. 3G). Furthermore, the angiogenesis around the ovarian graft showed a significant increase in the Fresh and FSH + S1P groups (red arrow points to the blood vessel). However, no obvious angiogenesis was observed in the other groups (Supplementary Fig. S1).

To substantiate whether FSH and S1P co-administration can restore the endocrine function of transplanted ovaries, we collected serum from mice at 7 days after transplantation to detect the secretion of E_2_, the levels of E_2_ in all three intervention groups were significantly higher than that in the PBS group (*P* < 0.05), and there was a significant difference between the FSH + S1P group and the FSH group in this respect (*P* < 0.05) (Fig. 3H). These data indicated that FSH and S1P co-administration promoted follicle survival and development, accelerated vascular angiogenesis and perfusion, and maintained their normal endocrine function in the transplanted ovaries.

### Molecular mechanism of FSH combined with S1P intervention to protect follicle survival in transplanted ovaries

To determine whether FSH promotes the production of endogenous S1P by up-regulating the expression of pSK-1 to maintain follicle survival in transplanted ovaries, we analyzed the production of S1P and pSK-1 proteins in ovaries at 24 h after transplantation by western blot assay. We found that the expression of S1P in the FSH + S1P group was remarkably higher than that in the PBS, FSH, and S1P groups (*P* < 0.01) (Fig. S2A, B). The expression of pSK-1 (a key enzyme for S1P production) in the FSH + S1P group was also significantly higher than that in the PBS, FSH, and S1P groups (*P* < 0.01) (Fig. S2C, D). These above results suggested that FSH and S1P co-intervention can increase the production of endogenous S1P by up-regulating the expression of pSK-1, to make up for the short half-life of S1P, so that S1P can continue to play its role.

To further clarify the intrinsic molecular mechanism of FSH and S1P co-intervention to protect the survival of follicles in transplanted ovaries, we treated human granulosa-like tumor cell line (KGN) with ischemia and hypoxia in vitro to simulate the state of ischemia and hypoxia in early transplanted ovaries and evaluated the protective effect of FSH and S1P co-intervention on ischemia-hypoxia-induced KGN cells. We found that KGN cells treated with different concentrations of CoCl_2_ and serum-free culture for 24 h showed a significant decrease in cell viability, of which the cell viability of the 400 µM CoCl_2_ treatment group was significantly lower than that of the Control group (*P* < 0.01) (Fig. S3A), so we chose 400 µM CoCl_2_ treatment for subsequent experiments. Then we added different concentrations of FSH, S1P and FSH combined with S1P to the cells when cells were in the state of ischemia and hypoxia, respectively. The optimal concentration of FSH, S1P, and FSH combined with S1P was selected according to cell viability and cell state. Finally, we chose 2.4 IU/mL FSH + 2 µM S1P for the follow-up experiment (Fig. S3B-D).

**Fig. 4:**
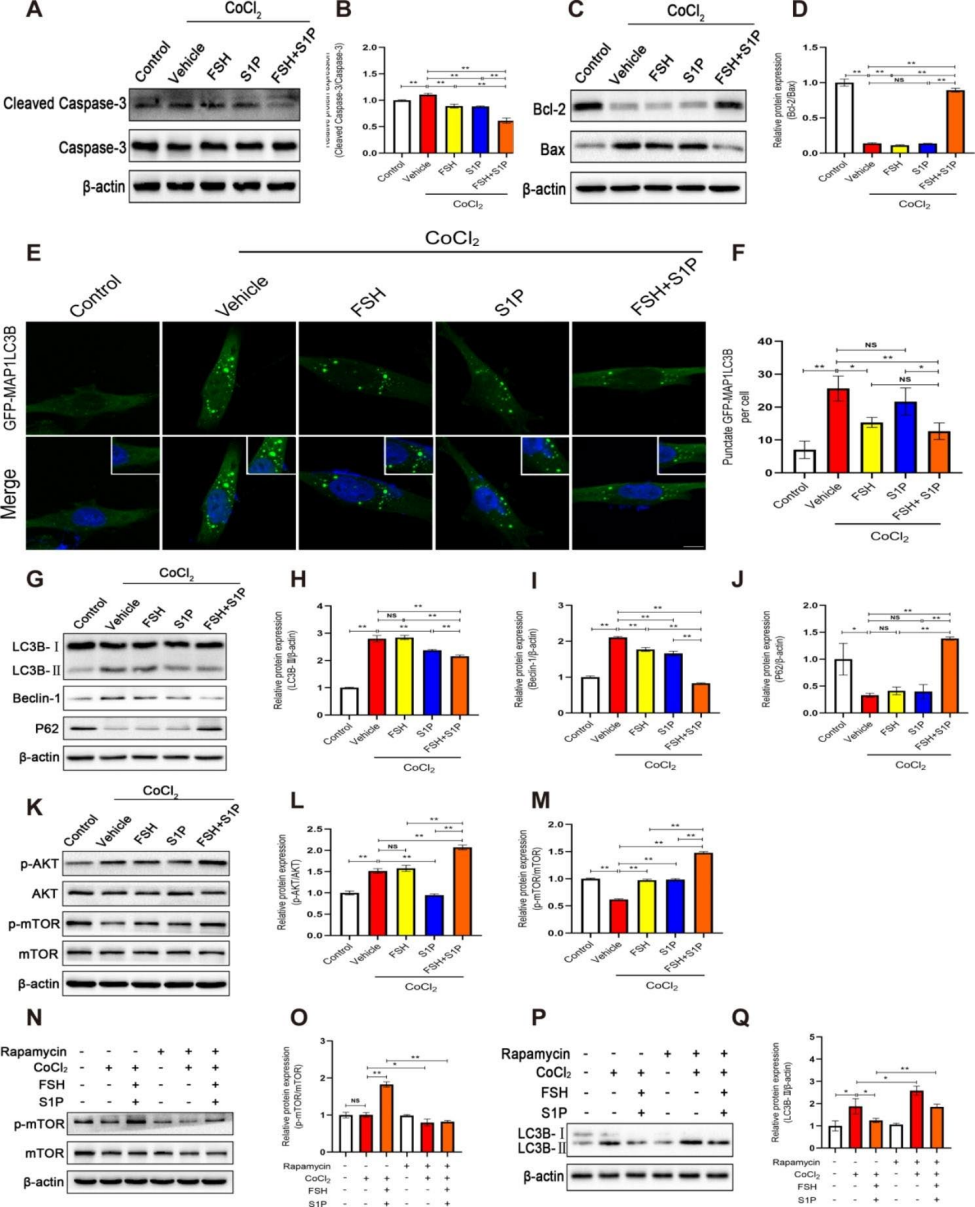
Molecular mechanism of FSH combined with S1P intervention to protect follicle survival in transplanted ovaries (**A**-**B**) Western blot analysis of the expression of Cleaved Caspase-3 in KGN cells treated with ischemia-hypoxia and its quantification. (**C**-**D**) Western blot analysis of the expression of Bcl-2 and Bax in KGN cells treated with ischemia-hypoxia and quantification Bcl-2/Bax ratio. (**E**) After KGN cells were transfected with GFP-MAP1LC3B plasmid, the production of autophagosomes in the cells was observed by the confocal laser scanning microscope (Scale bar: 10 μm). (**F**) Quantification of GFP-MAP1LC3B puncta per cell. (**G**-**J**) Western blot analysis of the expression of LC3B、Beclin-1 and P62 in KGN cells treated with ischemia-hypoxia and its quantification. (**K**-**M**) Western blot analysis of the expression of p-AKT and p-mTOR in KGN cells treated with ischemia-hypoxia and its quantification. (**N**-**O**) Western blot analysis of the expression of p-mTOR in KGN cells treated with the mTOR inhibitor Rapamycin. (**P**-**Q**) Western blot analysis of the expression of LC3B in KGN cells treated with Rapamycin. (*P < 0.05; **P < 0.01)

To evaluate whether FSH and S1P co-intervention can resist ischemia-hypoxia-induced KGN cells apoptosis, we performed TUNEL staining and detected the production of apoptosis-related proteins. TUNEL red fluorescence represents apoptotic cells. The apoptotic rates in the three intervention groups were significantly lower than those in the Vehicle group (*P* < 0.05). Among them, the apoptotic rate of the FSH + S1P group was the lowest, and there was a significant difference with the S1P group (*P* < 0.01), but no difference with the FSH group (*P* > 0.05) (Fig. S4A, B). Western blot results showed that the expression of Cleaved Caspase-3 was significantly up-regulated in ischemia-hypoxia-induced KGN cells, which was consistent with the results we observed in ovaries at 24 h after transplantation. Compared with the Vehicle, FSH, and S1P groups, the expression of Cleaved Caspase-3 in FSH + S1P group was significantly lower (*P* < 0.01) (Fig. 4A, B). The results of the Bcl-2/Bax ratio in the FSH + S1P group was significantly higher than that in the Vehicle, FSH, and S1P groups (*P* < 0.01) (Fig. 4C, D). Furthermore, to verify that FSH and S1P co-intervention can inhibit the excessive autophagy of ischemia-hypoxia-induced KGN cells, we transfected cells with a green fluorescent protein (GFP)-labeled microtubule-associated protein 1 light chain 3β (MAP1LC3B) expression plasmid (GFP-MAP1LC3B) to detect the formation of autophagosomes: GFP green fluorescent spots represent autophagosomes (Fig. 4E). We found that the production of autophagosomes in the Vehicle group was significantly higher than that in the Control group after ischemia-hypoxia treatment (*P* < 0.01), indicating that ischemia-hypoxia stress can induce autophagy in KGN cells, while FSH and S1P co-intervention could significantly inhibit the generation of autophagosomes (*P* < 0.01), meanwhile, there was a significant difference in the number of autophagosomes in the cells of the FSH + S1P group and the S1P group (*P* < 0.05) (Fig. 4F). The expression results of several autophagy-related proteins detected by western blot showed that ischemia-hypoxia treatment significantly increased the level of cellular autophagic protein LC3B-II, while FSH and S1P co-intervention seemed to inhibit the up-regulation of this autophagic protein (*P* < 0.01), compared with FSH and S1P groups, the inhibitory effect of FSH + S1P group was more significant (*P* < 0.01) (Fig. 4H). The expression of Beclin-1 in the FSH + S1P group was significantly lower than that in the Vehicle, FSH, and S1P groups (*P* < 0.01) (Fig. 4I). Additionally, the expression of P62 in the FSH + S1P group was significantly higher than that in the Vehicle, FSH, and S1P groups (*P* < 0.01) (Fig. 4J).

To verify the molecular mechanism of FSH and S1P co-administration to regulate autophagy in KGN cells, we detected the expression of major proteins in PI3K/AKT/mTOR, and further confirmed this pathway by adding mTOR inhibitor. The expression of p-AKT and p-mTOR in the FSH + S1P group of KGN cells were significantly higher than those in the Vehicle, FSH and S1P groups (*P* < 0.01) (Fig. 4K-M), We observed the same results in ovaries at 24 h after transplantation (Fig. S5). By adding the mTOR inhibitor Rapamycin, we found that the expression of LC3B-II was significantly increased in the FSH + S1P group, indicating that the FSH and S1P co-administration may inhibit the autophagy of KGN cells by promoting the phosphorylation of mTOR (Fig. 4N-Q). These results indicated that FSH and S1P co-administration can protect the survival of follicles in transplanted ovaries by inhibiting ovarian granulosa cell apoptosis and excessive autophagy under ischemia-hypoxic stress, and the mechanism of inhibiting autophagy may be by promoting the AKT/mTOR axis phosphorylation.

## Discussion

Freezing damage during ovarian vitrification, follicle losses due to ischemia and hypoxia during the initial stage of avascular anastomosis transplantation and egg development damage due to delayed angiogenesis are the “bottlenecks” that limit the general application of this technology [12]. FSH is an important hormone in follicle growth and development, and has been shown to reduce follicle loss during ovarian vitrification and transplantation, and increase angiogenesis [28, 29, 34]. S1P is used as an anti-apoptotic agent in ovarian vitrification and transplantation [35]; however, due to its short half-life, the protective effect cannot be produced continuously [36]. In the present study, we propose a novel role of FSH combined with S1P to intervene in the ovarian vitrification process to protect vitrification and transplanted ovaries from freezing and ischemic-hypoxic injury. Our findings showed that the combined application of FSH and S1P during ovarian vitrification not only protected the ovaries from vitrification cryopreservation induced injury, but also inhibits apoptosis and autophagy in transplanted ovaries, promotes follicle survival, enhances angiogenesis in transplanted ovaries, and restores the endocrine function of transplanted ovaries. Mechanistically, FSH promotes endogenous S1P production in the ovary through up-regulation of pSK-1 expression. In addition, combined with S1P, FSH inhibited ischemia-hypoxia-induced apoptosis of ovarian granulosa cells and suppressed granulosa cell autophagy by regulating the AKT/mTOR signaling pathway.

Follicle survival during ovarian cryopreservation is a prerequisite for the proper development of the transplanted ovary. Researchers have tried to add some protective factors such as luteinizing hormone (LH) [37], erythropoietin (EPO) [38], angiopoietin-2 (Ang-2) [39], FSH [29], resveratrol [40], and S1P [41] to ameliorate the damage caused by vitrificating and transplantation of ovaries during ovarian vitrification and transplantation. S1P is a catabolite of cell membrane sphingomyelin and plays an important role in regulating cell growth, proliferation, differentiation, and angiogenesis [42]. S1P is also widely used in the field of reproduction: in the study of resistance to chemotherapy-induced ovarian damage, it was found that S1P can inhibit the apoptosis of primordial follicles induced by chemotherapy drugs [20]. S1P in the ovary can promote the proliferation of granulosa cells and maintain the survival of follicles [43]. Guzel et al. found that S1P could reduce follicular atresia caused by cryogenic injury during cryopreservation of ovarian cortical samples and improve the survival rate of cryopreserved ovaries [44]. Nevertheless, high doses of S1P induce apoptosis of granulosa cells and induce follicular atresia [27]. Studies have demonstrated that S1P has paradoxical toxic effects, as microinjection of S1P in keratinocytes induces strong cell growth arrest and reduces cell proliferation [45]. In previous studies, FSH intervention improved follicle survival in frozen ovaries, but excessive concentrations of FSH led to excessive depletion of follicles in transplanted ovaries [28]. Therefore, we considered combining appropriate doses of FSH and S1P to intervene in the ovarian vitrification process to compensate for the inadequacy of the individual interventions. Our previous study, by screening different combined doses of FSH and S1P, has demonstrated that 0.3 IU/mL FSH combined with 2 µM S1P intervention can better protect the function of the cryopreservation ovaries than other combined intervention groups. Therefore, in this study, we will continue to use this combined concentration. We found that the intervention of 0.3 IU/mL FSH combined with 2 µM S1P during the whole process of ovarian vitrification can reduce the apoptosis of cryopreserved ovaries, inhibit follicular atresia. It can maintain a certain number of primordial follicles and avoid excessive activation of primordial follicles.

Studies have shown that a large number of follicles are lost after transplantation rather than during cryopreservation [13]. Follicle loss can be partly attributed to graft ischemia and hypoxia before new blood flow reestablishment [46]. Therefore, reducing the loss of follicles in the early stage of transplantation and promoting the angiogenesis of the graft become the key points to improving the survival rate of the graft. FSH can increase the blood supply of ovarian avascular transplantation by up-regulating the expressions of Cx43, Cx37, VEGF, and VEGFR2 [47]. Added 2 µM S1P during cryopreservation to maintain the primordial follicle pool in the post-transplant ovary [41]. Previous study found that the anti-Mullerian hormone (AMH) levels and the proportion of primordial follicles in the ovaries of the S1P-treated group were significantly higher than those of the untreated group [48]. Other study showed that the increase in vascular density of transplanted ovaries was dependent on S1P treatment, which accelerated angiogenesis while reducing ischemia-reperfusion injury and improved the success rate of human ovarian tissue xenografts [49]. However, Henry’s study showed that although the addition of S1P in sheep ovary cryopreservation and transportation can improve the quality of primordial follicles, with the extension of culture time, the short half-life of S1P cannot maintain a long-term effect, which will lead to the number of follicles and proliferating cells decreased sharply [36]. In addition, continuous S1P perfusion in vitro is expensive and not suitable for clinical use. Therefore, corresponding measures are needed to make S1P continue to function to fully exert its biological activity. Studies have shown that FSH and VEGF can promote endogenous S1P production in ovarian granulosa cells [27]. Therefore, we used the combination of FSH and S1P to stimulate endogenous S1P production to compensate for the short half-life of S1P. In theory, it can better protect ovarian function and improve the quality of follicles in transplanted ovaries. Our results showed that FSH can promote the production of S1P in ovarian and granulosa cells by up-regulating the expression of pSK-1. In the early stage of ovarian transplantation, FSH combined with S1P intervention could maintain a higher proportion of primordial follicles than the individual intervention and avoid over-activation of primordial follicles; although the proportion of primary follicles decreased in the FSH and S1P intervention group compared with the individual intervention group, it promoted the production of secondary follicles and antral follicles, and reduced ovarian granulosa cell apoptosis and thus inhibited follicular atresia, and inhibited excessive autophagy caused by ischemic and hypoxic stress in the ovaries. With the extension of transplantation time, FSH combined with S1P intervention could promote follicle development and maturation, accelerate the angiogenesis of transplanted ovaries by up-regulating the expression of VEGF, and maintain follicle survival by up-regulating the expression of Cx37 and Cx43, thus promoting the recovery of ovarian function. In summary, this combined intervention not only compensated for the shortcomings of individual interventions, but also showed better advantages than individual interventions during ovarian vitrification and transplantation.

Apoptosis and autophagy are the two main forms of cell death [50]. Granulosa cell apoptosis is usually considered to be the main cause of follicular atresia, but recent studies have also supported the fact that autophagy can cause follicular atresia [51]. Autophagy is a degradation/recycling system that exists in eukaryotic cells to maintain cellular metabolism and internal homeostasis [52]. Moderate autophagy helps to maintain the number of primordial follicles and oocyte quality; however, under stimuli such as ischemia and hypoxia, nutrient deprivation, and oxidative stress, cells will undergo excessive autophagy leading to impaired follicular development, which in turn affects female reproduction [51]. In the present study, we evaluated whether FSH combined with S1P protects follicle survival in transplanted ovaries by inhibiting excessive autophagy induced by ischemic-hypoxic stress. Shen et al. found that FSH inhibits autophagy by activating the PI3K-AKT-mTOR signaling cascade, down-regulating FOXO1 transcriptional activity and FOXO1 deacetylation, and protecting ovarian granulosa cells from oxidative stress damage [30]. Another study showed that FSH inhibits supporting cellular autophagy by activating the PI3K-AKT-mTOR pathway, reducing lysosomal biogenesis, and inhibiting TFEB nuclear translocation [53]. The high mTOR activity under FSH stimulation may prevent the activation of Ulk1 by phosphorylating ULK1 Ser 757, thereby inhibiting autophagy [54]. Sphingolipid ceramides and S1P constitute a “rheostat system” in which ceramide promotes cell death and S1P increases cell survival, and together they control various cellular functions, including proliferation, cell death, and autophagy [55]. During nutrient starvation, elevated S1P levels following increased SK-1 activity can induce protective autophagy, and this S1P-induced autophagy acts dependently on the PI3K pathway, either directly or indirectly, and is associated with inhibition of phosphorylation of mTOR substrates (p70S6K, 4E-BP1) and a modest increase in Beclin-1 amounts, thereby sparing cells with apoptotic features death. Ceramide acts upstream of S1P by inhibiting phosphorylation of AKT/PKB [31]. Ceramides have been reported to inactivate the mTOR pathway or dissociate the Beclin1-Bcl-2 complex, leading to autophagy [56, 57]. S1P treatment was able to inhibit amino acid deprivation (AA(-)) and C2-ceramide-induced autophagy, which was related to the activation of the mTOR pathway by S1P through the S1P receptor [58]. These results suggest that autophagy may be a novel function of S1P in cell survival. In our study, AKT and mTOR phosphorylation levels were increased in ovarian and granulosa cells under ischemic hypoxic stress after FSH combined with S1P treatment, suggesting that our cellular signals generated by the different effects of FSH and S1P usually target the mTOR pathway, which may be related to the inhibition of mTOR dephosphorylation caused by high levels of ceramide in the stress state. It is suggested that FSH combined with S1P may counteract the occurrence of autophagy by activating the AKT/mTOR pathway and protect cells from excessive autophagy due to ischemia and hypoxia.

In summary, our results suggest that FSH combined with S1P intervention could inhibit apoptosis and autophagy in vitrification freeze-thaw and transplanted ovaries promoted follicle survival, enhance angiogenesis in transplanted ovaries, and restored the endocrine function of transplanted ovaries. Exploration of the mechanism revealed that FSH promoted endogenous S1P production in ovaries by up-regulating pSK-1 expression, and FSH combined with S1P inhibited ischemia-hypoxia-induced apoptosis of ovarian granulosa cells and suppressed granulosa cell autophagy by regulating AKT/mTOR signaling pathway. The study provides a scientific basis for further improving the efficacy and safety of vitrification freeze-thaw ovarian transplantation.

### Electronic supplementary material

Below is the link to the electronic supplementary material.

**Supplementary Material 1**

## Data Availability

The datasets used or analysed during the current study are available from the corresponding author on reasonable request.
