# Supplementary material for: Roles of follicle stimulating hormone and sphingosine 1-phosphate co-administered in the process in mouse ovarian vitrification and transplantation
Source: J Ovarian Res. 2023 Aug 24;16:173. doi: 10.1186/s13048-023-01206-1 (PMC10463983; doi:10.1186/s13048-023-01206-1)
Supplement: Supplementary file 1 — Supplementary Material 1 [file 13048_2023_1206_MOESM1_ESM.docx]

**
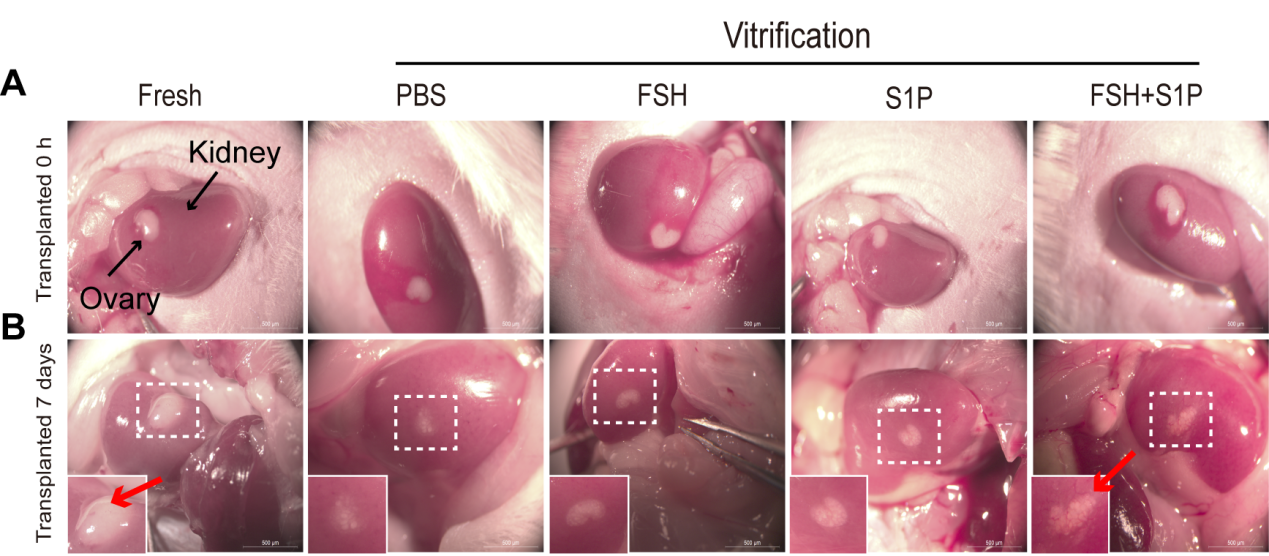
**

**Fig.S1 Representative images of the ovaries at 7 days after transplantation**

(A) Ovaries from 21-day-old mice were transplanted under the renal capsule of 6-8 week old recipient mice (Scale bars: 500 μm). (B) The images of ovaries at 7 days after transplantation, red arrows indicate neovascularization (Scale bars: 500 μm).


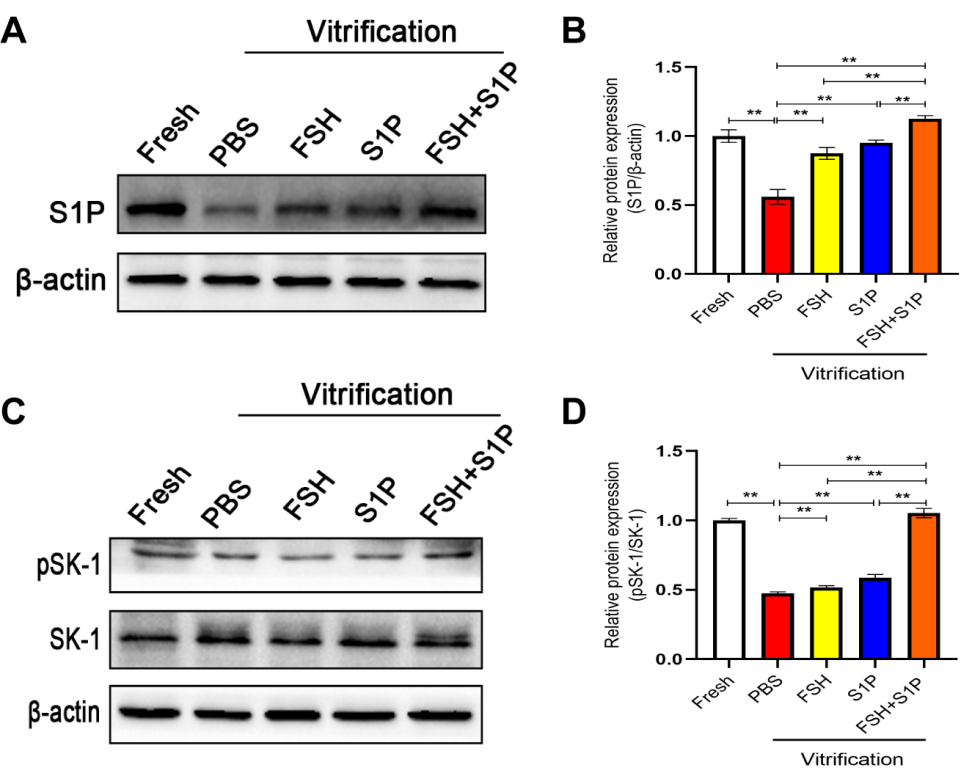


**Fig.S2 Mechanism by which FSH promotes S1P production in the ovaries at 24 h after transplantation**

(A-B) Western blot analysis of the expression of S1P of ovaries at 24 h after transplantation and its quantification. (C-D) Western blot analysis of the expression of pSK-1 of ovaries at 24 h after transplantation and its quantification. (**P*<0.05 ; ***P*<0.01).
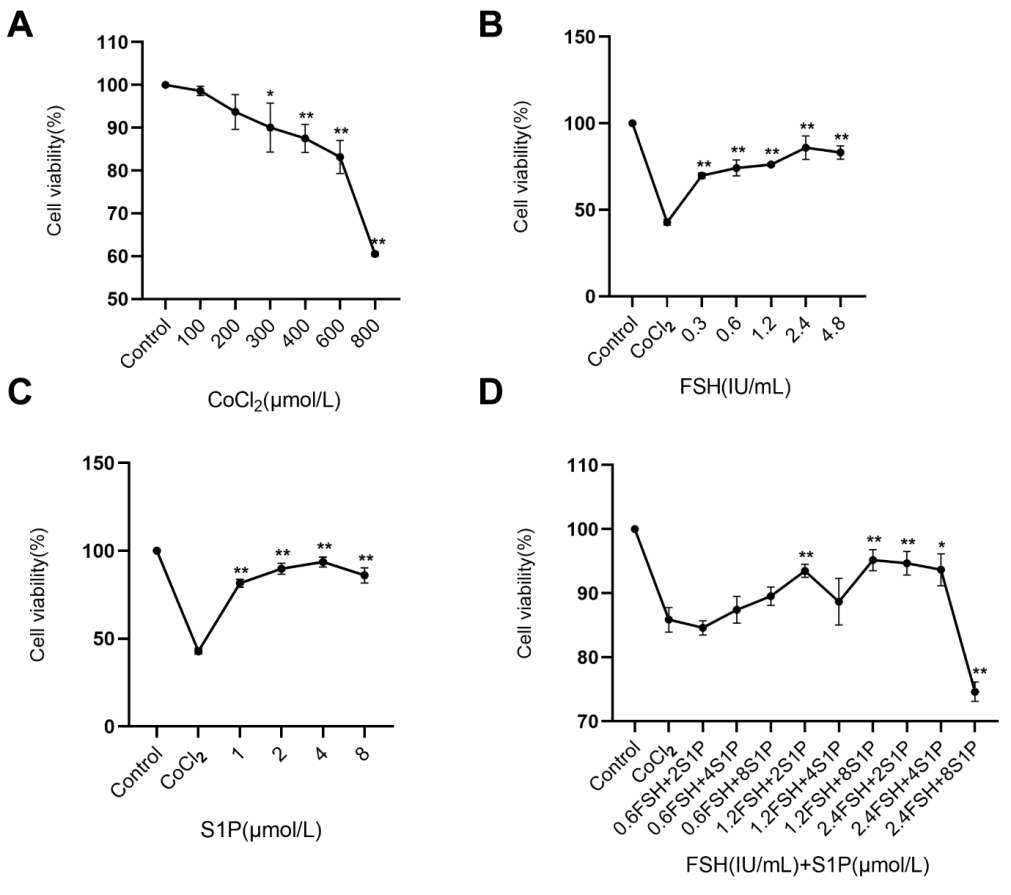


**Fig.S3 Effects of FSH and S1P co-intervention on the viability of KGN cells under ischemia and hypoxia**

(A) The KGN cells were treated with different concentrations of CoCl_2_ for 24 h, and cell viability was then determined using the CCK-8 assay. (B) The KGN cells were treated with 400 μM CoCl_2_ and different concentrations of FSH for 24 h, and cell viability was determined using the CCK-8 assay. (C) The KGN cells were treated with 400 μM CoCl_2_ and different concentrations of S1P for 24 h, and cell viability was determined using the CCK-8 assay. (D) The KGN cells were treated with 400 μM CoCl_2_ and different concentrations of FSH and S1P for 24 h, and cell viability was determined using the CCK-8 assay. (**P*<0.05 ; ***P*<0.01).


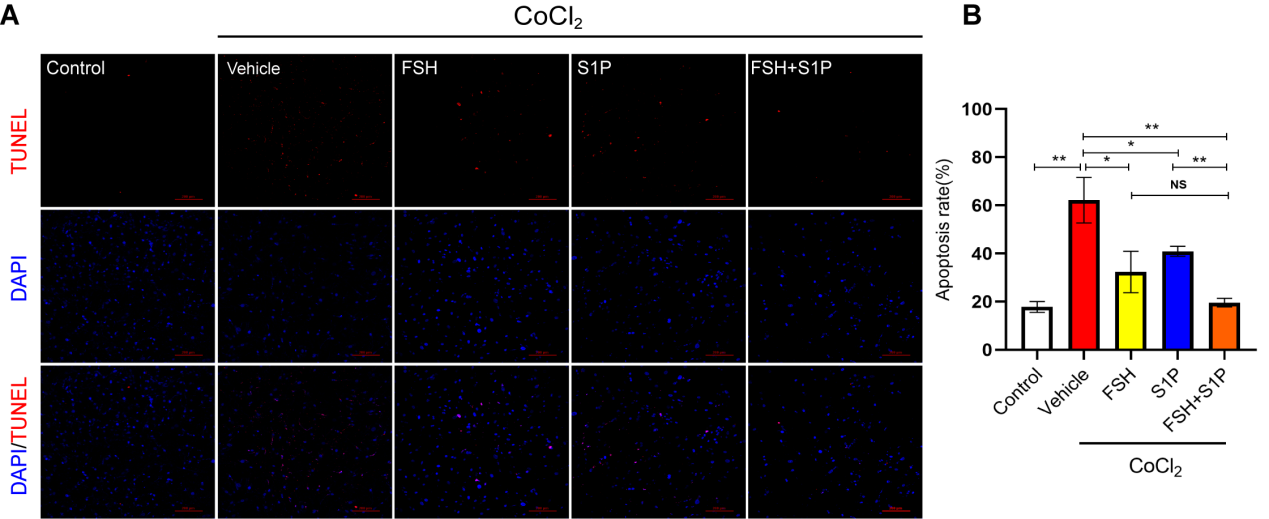


**Fig.S4 FSH and S1P co-intervention inhibits apoptosis of KGN cells**

(A) TUNEL staining to detect KGN cells apoptosis (Scale bar: 200 μm). (B) Statistical analysis of apoptotic rate. (**P*<0.05 ; ***P*<0.01).
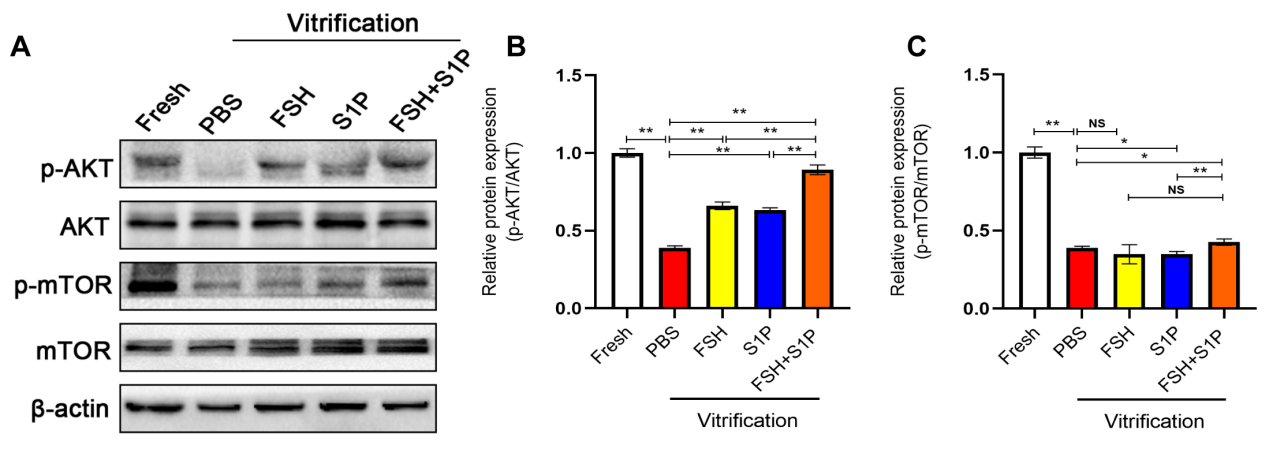


**Fig.S5 FSH and S1P co-intervention inhibits autophagy of ovaries at 24 h after transplantation by up-regulating AKT/mTOR signaling pathway**

(A-C) Western blot analysis of the expression of p-AKT and p-mTOR of ovaries at 24 h after transplantation and its quantification. (**P*<0.05 ; ***P*<0.01).

**Materials and Methods**

**TUNEL assay**

Apoptosis was determined using the in situ terminal deoxynucleotidyl

transferase-mediated nick end labelling (TUNEL) assay (Vazyme, A113-02), then the sections were re-stained with 2 μg/mL DAPI (Solarbio, C0065) at room temperature for 5 min. And the apoptotic rate was analyzed using Image-Pro Plus 6.0 software. Randomly selected three areas of the slice/cell climbing slice to take pictures and record, and calculated the percentage of TUNEL-positive cells (mainly granulosa cells) in each area to the total number of cells, which is the apoptotic rate.

**Immunohistochemistry**

Paraffin-embedded ovaries were cut into serial sections, the sections were dewaxed and dehydrated, and antigen repair was performed in sodium citrate buffer, the samples were repaired twice in a microwave at 100℃ for 15 min each time. The sections were incubated with 3% H_2_O_2_ for 10 min to eliminate endogenous peroxidase activity. After being washed with PBS, the sections were blocked with 10% goat serum (Beyotime, C0265) for 30 min at 37℃. Following blocking, the primary antibodies of Cx37 (Abcam, ab181701, 1:200), Cx43 (Abcam, ab11370, 1:200), and VEGF (Abcam, ab52917, 1:200) were added to tissues and incubated overnight at 4℃. The negative control was replaced by PBS. The sections were washed three times with PBS and incubated with HRP-conjugated IgG secondary antibody (rabbit: ZSGB-BIO, ZB-2301, 1:500) at 37℃ for 60 min, and the sections were stained with DAB (ZSGB-BIO, ZLI-9018) for 30 s. After re-staining with hematoxylin, the sections were dehydrated, transparent, and sealed. The photographs were taken in the bright field with DP Controller 3.1.1.267 image acquisition system and used Image-Pro Plus 6.0 software to count% of DAB positive area.

**Immunofluorescence**

The ovaries were made into frozen sections. The frozen sections were washed twice with PBS buffer after dried at room temperature for 30 min, permeabilized with 0.5% TritonX-100 at room temperature for 30 min, and blocked with 10% goat serum at room temperature for 1 h. Following blocking, sections were incubated overnight with CD31 (Abcam, Ab28364, 1:50) primary antibody at 4℃. The next day, the sections were incubated with the goat anti-rabbit fluorescent secondary antibody (Invitrogen, A11010, 1:500) at room temperature for 2 h after being washed with PBST buffer. The sections were sealed with an anti-fluorescence quenching agent after re-stained with 2 μg/mL DAPI solution, observed by confocal laser scanning microscope and imaged. Image-Pro Plus 6.0 software was used for analysis.

**Serum E_2_ levels**

Whole blood was collected from the mice at 7 days after transplantation, the serum was collected after centrifugation at 2000 rpm/min for 20 min, an enzyme-linked immunosorbent assay (ELISA) was performed to analyze E_2_ (Mlbio, ml063198-2) in the serum. Set the blank hole to zero, and measure the absorbance at 450 nm (OD value). The standard curve was drawn with the standard concentration as the abscissa and the OD value as the ordinate, and calculate the concentration of E_2_ in serum according to the standard curve. The minimum concentration of E_2_ in mice was less than 0.1 pg/mL.

**Cell viability analysis**

The cell viability was determined by using Cell Counting Kit-8 (APExBIO, K1018). Our experimental procedures were performed according to the manufacturer’s instructions. In brief, KGN cells were plated into 96-well plates at a concentration of 8×10^3^ cells/well, grown to 80%, and exposed to the indicated treatments. CCK-8 solution (10 μL) was added to each well, followed by incubation for 2 h at 37°C. Then measure the absorbance at 450 nm (OD value).

**Measurement of autophagosome formation**

KGN cells were seeded on coverslips in 24-well plates, and grown to 70% to 80% confluency before transfection with the green fluorescent protein (GFP)-tagged microtubule-associated protein 1 light chain 3 β (MAP1LC3B)

expression plasmid (GFP-MAP1LC3B) (GeneChem Co., Ltd, NM_022818), 48 h later, cells were exposed to the desired experimental treatment, then the cells were washed three times with PBS and re-stained with DAPI. GFP-MAP1LC3B spots in the cytoplasm were observed under the confocal laser scanning microscope. A total of three replicates of the experiment were performed, and three areas were randomly selected in each cell crawl, and the number of spotted GFP-MAP1LC3B was counted and plotted in a histogram using graphing software (Graph Pad).

**The preparation of the vitrification and thawing solution**

1.Basic culture medium (50 mL) : DMEM/F-12 medium (49 mL) (Hyclone)+1% penicillin-streptomycin (0.5 mL) (Meilunbio, PWL062)+1% dimethyl sulfoxide (DMSO) (0.5 mL) (MP Biomedicals, 68-67-5), store at 4°C.

2. Culture solution (50 mL) :10% Bovine Serum Albumin (BSA) (5 g) (Sigma, 9048-46-8) +basic culture medium, dilute to 50 mL after dissolving, store at -20°C.

3. Preequilibrated solution (50 mL) : 20% BSA (10 g)+ethylene glycol (EG) (8.36 mL)+basic culture medium, dilute to 50 mL after dissolving, store at -20°C.

4. Vitrification solution (50 mL): 20% BSA (10 g)+30% polysucrose (15 g)+ sucrose (8.56 g)+EG (15.3 mL)+basic culture medium, dilute to 50 mL after dissolving, store at -20°C.

5. 0.5 mol/L thawing solution Ⅰ (50 mL): sucrose (8.56 g)+20% BSA (10 g) +basic culture medium, dilute to 50 mL after dissolving, store at -20°C.

6. 0.25 mol/L thawing solution Ⅱ (50 mL): 0.5 mol/L thawing solution Ⅰ (25 mL) +basic culture medium (25 mL), store at -20°C.

7. 0.125 mol/L thawing solution Ⅲ (50 mL): 0.25 mol/L thawing solution Ⅱ (25 mL) +basic culture medium (25 mL), store at -20°C.

**Primary Antibodies of Western Blotting**

Bcl-2 (Affinity, AF6139, 1:1000), Bax (CST, 2772S, 1:1000), Cleaved Caspase-3 (CST, 9661S, 1:1000), Caspase-3 (CST, 9662S, 1:1000), pSK-1 (Proteintech, 19561-1-AP, 1:500), SK-1 (Bioss, bs-2652R, 1:500), S1P (Abcam, ab59870, 1:1000), LC3B (CST, 43566,1:1000), P62 (Abcam, ab56416, 1:1000), Beclin-1 (CST, 3495P,1:1000), AKT (Proteintech, 60203-2-Ig, 1:1000), p-AKT (CST, 4060, 1:1000), mTOR (Proteintech, 66888-1-Ig, 1:1000), p-mTOR (CST, 2971, 1:1000), β-actin (Bioss, bs-0061R, 1:1000).
